# Change in Hemoglobin Levels due to Anesthesia in Mice: An Important Confounder in Studies on Hematopoietic Drugs

**DOI:** 10.1007/s12575-009-9018-8

**Published:** 2009-12-03

**Authors:** Anita Gothelf, Pernille Hojman, Julie Gehl

**Affiliations:** 1 Department of Oncology 54O5, Copenhagen University Hospital Herlev, 75 Herlev Ringvej, 2730, Herlev, Denmark; 2 Centre of Inflammation and Metabolism, Copenhagen University Hospital, 9 Blegdamsvej, 2100, Copenhagen, Denmark; 3 Department of Oncology 54B1, Copenhagen University Hospital Herlev, 75 Herlev Ringvej, 2730, Herlev, Denmark

**Keywords:** anesthesia, hemoglobin, in vivo, fentanyl, midazolam

## Abstract

Analgesic and anesthetic drugs may have an impact on the results achieved from animal experiments. In the study presented here, we try to enlighten whether anesthesia with fentanyl/fluniasone and midazolam (Hypnorm and Dormicum) has an influence on measurements of hemoglobin in mice. In a cross-over study, we have compared hemoglobin levels in two groups of mice: anesthetized versus non-anesthetized and found significant decrease in hemoglobin levels in the anesthetized group (*p* < 0.05) unrelated to which group received the anesthesia. The mean hemoglobin levels after intraperitoneal administration of Hypnorm and Dormicum was 8.7 mmol/L compared to mean hemoglobin 9.9 mmol/L before anesthesia (*p* < 0.001), and the decrease lasted for more than 30 min. These results show that anesthesia can be an important confounder in studies involving measurements of hemoglobin, and this should be taken into account when planning studies and analyzing data.

## 1. Introduction

When performing studies involving laboratory animals, appropriate anesthesia is warranted although certain drugs may influence on the results.

It has been shown that anesthetized mice have a decreased respiratory rate and may develop hypoxia, hypercapnia, and acidosis [1], all of which theoretically can affect the data depending of aim and design of the study.

In our unit, we use the combination of Hypnorm (fentanyl/fluniasone), which is a neuroleptanalgesia, and a sedative Dormicum (midazolam). This combination provides sufficient degree of anesthesia and analgesia but has side effects as shown by Whelan et al. [1].

During studies with gene electrotransfer to muscle [2,3] and skin (Gothelf et al, submitted for publication) with erythropoietin (EPO) involving sequential blood sampling over time, we have discovered some inconsistency regarding the blood samples. At the end of the study, an unexpected decrease in hemoglobin levels compared to earlier levels was observed, the only obvious difference being that the animals were anesthetized.

We thus planned two studies to enlighten whether the anesthesia has an influence on the measurement of hemoglobin.

## 2. Material and Methods

### 2.1. Animals

Female NMRI mice (own breed, Copenhagen University Hospital Herlev), 15 weeks old, were used in this study. The animals were kept in a pathogen-free environment with 12 h/12 h light/darkness cycle and food and water ad libitum. The study was performed with permission from the Danish Animal Experiments Inspectorate and in accordance with the European Convention for the Protection of Vertebrate Animals used for Experimentations.

All mice were humanely euthanized with quick cervical dislocation at the termination of the study.

### 2.2. Anesthesia

When applied, the anesthesia was Hypnorm (0.4 ml/kg, Janssen Saunderton, Buckinghamshire, England) and Dormicum (2 mg/kg, Roche, Basel, Switzerland) diluted in sterile water. The anesthesia was administered as intraperitoneal injections.

### 2.3. Measurement of the Hemoglobin Levels

Hemoglobin was analyzed in millimole per liter from blood samples using a HemoCue Hb201+ (HemoCue, AB, Sweden). A hematocrit straw was inserted in the medial angle of the eye, directed along the bony wall and the plexus retro-orbitalis was punctured. A drop of blood was placed on a HemoCue slide and analyzed immediately.

### 2.4. Design of Cross-Over Study

The design of the study is visualized in Table 1. Twelve mice were divided in two groups A and B. Blood samples were collected at day 0 and from then once a week. The first four blood samples were collected during anesthesia in group A and without anesthesia in group B. From the fifth blood sample and to termination of the study, blood samples were collected without anesthesia in group A but during anesthesia in group B.

**Table 1:** The design of the cross-over study

Blood sample Number (days)	Group A (*n* = 6)	Group B (*n* = 6)
1 (0)	+ anesthesia	- anesthesia
2 (7)		
3 (14)		
4 (21)		
5 (28)	- anesthesia	+ anesthesia
6 (35)		
7 (42)		
8 (49)		
9 (63)		

Group A had the first four blood samples collected during anesthesia, whereas group B served as controls. From sample number 5 and to the end of the study group B received anesthesia and group A served as controls

### 2.5. Design of the Time-Course Study

To investigate the levels of hemoglobin over time in anesthetized and non-anesthetized animals, a time-course study was performed. Mice were randomly divided in two groups and had pre-study values of hemoglobin measured at time *t* = -10 min. Group 1 (*N* = 8) was subsequently anesthetized as previously described, whereas group 2 (*N* = 6) received no anesthesia. The injection of anesthesia was considered as *t* = 0 min, and blood samples were collected from both groups at *t* = 10, 20, and 30 min.

An additional study with the same set-up but with continued blood sampling to *t* = 105 min after injection of anesthesia was carried out to validate the results (*N* = 6).

### 2.6. Statistical Methods

Data from the cross-over study and from the time-course study was analyzed using a repeated measurements model (statistical software SAS version 9.1). In the time-course study, pre-study hemoglobin level was regarded as baseline. *P* values < 0.05 were considered significant.

## 3. Results

### 3.1. Cross-Over Study

**Figure 1:**
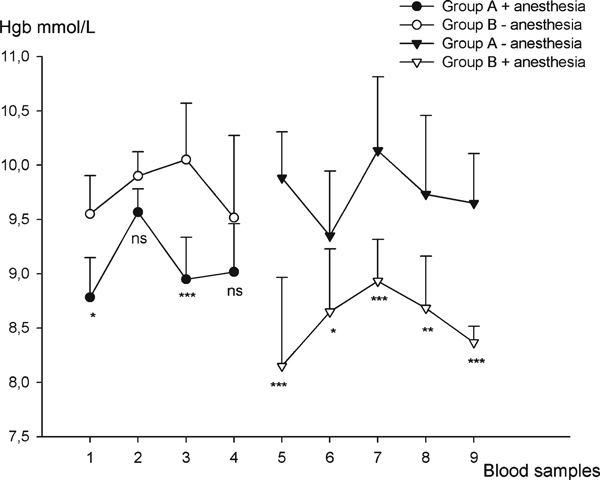
**Illustration of the hemoglobin measurements (cross-over study)**. At blood samples 1–4, group A received anesthesia and group B served as control. At blood samples 5–9, group A served as control and group B received anesthesia. The mean values + SD are presented. *P < 0.05, **P < 0.01, ***P < 0.001.

In Figure 1, the results from the cross-over study are depicted. At the first four measurements of hemoglobin, group A received anesthesia prior to the blood sampling and had significantly lower hemoglobin levels compared to group B, which received no anesthesia (*p* < 0.05, and *p* < 0.001 at blood samples 1 and 3, respectively). From blood sample 5 and to the termination of the study, the treatments were crossed over, and group B received anesthesia prior to the blood sampling. At blood samples 5–9, group B had significantly lower hemoglobin compared to group A (*p* < 0.001, 0.05, 0.001, 0.05, and 0.001, respectively). The drop in hemoglobin in group B + anesthesia at blood sample 5 is mainly due to a single mouse with hemoglobin of 6.4 mmol/L (mean, 8.15 mmol/L).

### 3.2. Time-Course Study

**Figure 2:**
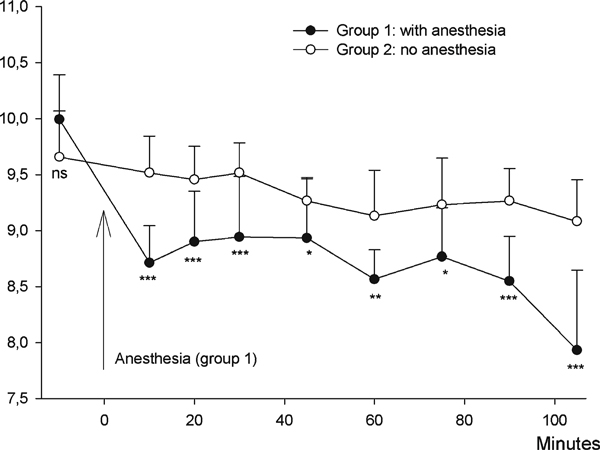
**Repeated blood samples over time (time-course study)**. Initially, both groups had blood samples collected without anesthesia (time = -10 min), and no significant difference was observed. At time = 0, group 1 received intraperitoneal injection of Hypnom/Dormicum. Subsequently, hemoglobin was measured at different time points, and the anesthetized group (group 1) had hemoglobin levels significantly lower than the non-anesthetized mice (group 2). *P < 0.05, **P < 0.01, ***P < 0.001.

At baseline (pre-study blood sample, *t* = -10 min), there was no significant difference between the anesthetized (group 1) and the control group (group 2; Figure 2). At the time point *t* = 10, 20, and 30 min, the difference between the groups was significant with significance levels of *p* < 0.001.

The mean hemoglobin in group 1 was 9.9 mmol/L prior to the anesthesia and decreased to 8.7 mmol/L 10 min after administration of the anesthesia (*p* < 0.001). At *t* = 20, 30, and 45 min, it remained at approximately 8.9 but decreased further and was 7.9 mmol/L at termination of the study. Group 2, which received no anesthesia, remained at the same level of hemoglobin (mean 9.5 mmol/L) at *t* = 10, 20, and 30 min but decreased slightly at *t* = 45 min and fell to 9.1 mmol/L at *t* = 105 min.

## 4. Discussion

Anesthesia induces physiological changes in animals as well as in humans. After administration of fentanyl or midazolam sedation, hypotension, bradycardia, vasodilatation, respiratory depression, and consequently hypoxia can occur [1,4].

This study shows that the hemoglobin level is significantly lower in anesthetized animals compared to non-anesthetized animals. The difference is due to the administered drugs and not to the mice or the blood sampling procedure, since the curves cross each other when the anesthetized group becomes non-anesthetized and vice versa. Throughout the study, we have collected all blood samples from the same anatomical site knowing that also different anatomical locations can give different hemoglobin levels [5].

In the second part of the cross-over study, the difference between the anesthetized and the non-anesthetized groups seems bigger than the difference in the first part of the study. This can, at some points, be explained by mice that differ considerably in hemoglobin from the mean values as in blood sample 5, but another explanation could be an impact on the mouse of the repeated blood sampling. Even though there is 1 week between each sampling, some extent of extra bleeding might occur. The decline in both curves at blood sample 8 and 9 could be in accordance with this theory.

That the difference between anesthetized and non-anesthetized is bigger after the cross-over of the treatment could be due to group A being artificially high. During anesthesia, the mice get hypoxic, and this is known to induce an endogenous release of EPO [6]. The main role of EPO is to stimulate the erythropoiesis in the bone marrow in a number of physiological conditions, and an increase in serum EPO has been observed 4 h after administration of Hypnorm/Dormicum to mice [1] (Gothelf et al., submitted for publication).

After induction of anesthesia, a decrease of approximately 1 mmol/L in hemoglobin occurs within 10 min and is not returned to the initial level within 30 min. Ceylan et al. [7] found a decrease in hemoglobin 30 min after intramuscular injection of tiletamin and zolazepam to sheep, but the level was normalized after 60 min. In the time-course study, the hemoglobin did not return to baseline within the 105 min the study lasted; on the contrary, the hemoglobin continued to decrease, and after 30 min, a decrease was also observed in the non-anesthetized group. This is probably due to the repeated blood sampling and handling of the animals.

The observed decrease in hemoglobin during anesthesia can be due to erythrocyte sequestration in the spleen [8], hypothermia, which is frequently occurring during anesthesia [9], or merely due to the analysis of hypoxic blood, but further studies are warranted in order to investigate the mechanism.

In this study, we have shown that anesthesia is an important confounder in the measurement of hemoglobin and must be taken into account when designing studies involving hemoglobin as a parameter.
